# Maximal accelerations for twelve weeks elicit improvement in a single out of a collection of cycling performance indicators in trained cyclists

**DOI:** 10.3389/fspor.2022.1027787

**Published:** 2023-01-10

**Authors:** Magnus K. Hyttel, Mathias Kristiansen, Ernst A. Hansen

**Affiliations:** ^1^ Sport Sciences – Performance and Technology, Department of Health Science and Technology, Aalborg University, Aalborg, Denmark; ^2^ Centre for Nutrition, Rehabilitation and Midwifery, University College Absalon, Slagelse, Denmark

**Keywords:** anaerobic threshold, cycling, exercise, incremental test, lactate threshold, pedal force, rate of perceived exertion, training

## Abstract

**Introduction:**

Cycling is a time-consuming sport. Cyclists, as many other athletes, therefore, focus on training effectively. The hypothesis was tested that twelve weeks of supplementary maximal acceleration training caused more favourable changes in cycling performance indicators as compared to changes measured in comparable control cyclists.

**Methods:**

Trained cyclists (*n *= 24) participated. A control group and a group performing maximal acceleration training, as a supplement to their usual training, were formed. The maximal acceleration training consisted of series of ten repetitions of outdoor brief maximal accelerations, which were initiated from low speed and performed in a large gear ratio. The cyclists in the control group performed their usual training. Performance indicators, in form of peak power output in a 7-s maximal isokinetic sprint test, maximal aerobic power output in a graded test, and submaximal power output at a predetermined blood lactate concentration of 2.5 mmol L^−1^ in a graded test were measured before and after the intervention.

**Results:**

Peak power output in the sprint test was increased (4.1% from before to after the intervention) to a larger extent (*p *= 0.045) in the cyclists who had performed the maximal acceleration training than in the control cyclists (−2.8%). Changes in maximal aerobic power output and in submaximal power output at a blood lactate concentration of 2.5 mmol L^−1^ were not significantly different between the groups (*p *> 0.351).

**Discussion:**

The results indicated that the applied supplementary maximal acceleration training caused modest favourable changes of performance indicators, as compared to the changes measured in a group of comparable control cyclists.

## Introduction

Cycling is a time-consuming sport, in comparison to other endurance sports. As an example, professional road cyclists may ride 30,000 to 35,000 km per year, which corresponds to more than 19 h of cycling per week. For comparison, elite marathon runners will likely never run more than 15 h per week (1). One of the consequences of the large number of hours performed in the sport of cycling, may be that many cyclists have a great focus on training effectively. In other words, they might want to achieve as large a performance enhancing effect as possible from the time they invest in their training. To succeed with that, quality of the training might be a key.

One of the aspects of quality of training is intensity. Studies have been performed to investigate the effect of intensified training on performance indicators in cyclists. For instance, it has been reported that short intervals (of 30-s duration) vs. effort-matched, more traditional, long intervals (of 5-min duration) resulted in larger improvements of performance indicators in cyclists (2, 3). Performance indicators are in this context for example peak power output in sprint cycling, maximal aerobic power output in a graded test, and submaximal power output at a predetermined blood lactate concentration [(Lā)].

Supplementing the usual cycling training with heavy strength training has also been shown to improve performance indicators in well-trained cyclists (4, 5). Lastly, a report by Koninckx et al. (2010) has indicated that brief maximal accelerations might have a performance enhancing effect in cyclists. Thus, Koninckx et al. (2010) studied twenty trained male cyclists with an average of six years of experience, and an average of 7,111 km cycled per year, during those years. The cyclists performed a twelve-week training intervention during the offseason, following a three-week rest period. The cyclists were divided (matched pairs) into two groups. One group performed conventional strength training (three sets of half squat and leg-press exercises at 15RM to 8RM) for leg-extensor muscles while the other group performed maximal-effort isokinetic ergometer cycling in four to eight bouts of twelve crank revolutions at 775 to 875 W at 80 rpm. Both groups performed the specific training twice per week. The two training groups increased average power output by a similar magnitude (5% to 8%) in a 30-min endurance performance test. In line with that, lactate-threshold power output and maximal power output obtained in a graded test were increased to similar extents for both groups (6).

Among the possible mechanisms responsible for the performance enhancement induced by strength training is increased pedalling effectiveness (7). Furthermore, physiological adaptations as increased force production capability of type I muscle fibres and less reliance on, less efficient, type II fibre recruitment (8–10) have been suggested (5).

A special type of cycling training that shares aspects with strength training is, according to anecdotes from the cycling community, recommended by coaches and applied by competitive cyclists. The special type of training consists of a series of brief maximal accelerations performed on the bicycle. The accelerations are commenced from a low speed, with the gearing set to a large gear ratio. This outdoor type of training is for example termed “functional strength training”, due to the large pedal force produced, especially in the initial phase of the accelerations. However, the effect of this type of training on performance indicators has not been systematically studied in a research study – before the present study. Consequently, even that the training presumably has been assessed as valuable by some cyclists, it is unknown whether it is sufficiently favourable for groups of cyclists, to be generally recommended.

Therefore, the purpose of the present study was to test the hypothesis that twelve weeks of supplementary maximal acceleration training*,* performed by trained cyclists, resulted in more favourable changes of performance indicators as compared to changes measured in comparable control individuals.

## Materials and methods

### Experimental design

The present study was an intervention study with cyclists participating in pre- and post-tests, which were separated by a twelve-week period. The pretests were conducted from the end of February until mid-March and consisted of two test sessions, performed on separate days. One test session consisted of assessment of body composition. Another test session consisted of a cycling test for assessment of cycling performance indicators and supplementary measurements including pedal force profile characteristics. Following the pretests, cyclists were allocated to either an intervention group (INT; *n* = 16) or a control group (CON; *n* = 14). To obtain homogeneous groups, the allocation aimed at matching the groups on training history, body composition, and cycling performance indicators. During the twelve-week period, cyclists in INT performed maximal acceleration training in addition to their usual cycling training. For comparison, cyclists in CON simply continued their usual cycling training. Posttests, which were identical to the pretests, were conducted from the beginning of June until mid-June. Due to some participants withdrawing during the study, both INT and CON were eventually amounting to *n *= 12.

### Participants

Thirty healthy male cyclists volunteered to participate in the present study. During the study, 6 cyclists withdrew due to injury (not related to the experiments, *n* = 4) or due to undisclosed reasons (*n* = 2). Thus, a total of 24 cyclists (mean ± SD: 42.9 ± 8.1 years, 81.2 ± 11.5 kg, 1.81 ± 0.06 m) completed the study. The cyclists had a background of 14.7 ± 9.5 years of cycling training. During the month prior to the initiation of the study, cyclists had completed 4.0 ± 1.6 training sessions per week, which resulted in 7.2 ± 3.1 h of training per week. According to previously published criteria, the cyclists in the present study could be categorised as “trained” to “well-trained” road cyclists (11). The cyclists had not completed conventional strength training three months prior to the study. Before participation, cyclists were informed of the purpose as well as the procedures of the study. Written consent was obtained from each participant. The study conformed to the standards set by the Declaration of Helsinki. The study was approved by The North Denmark Region Committee on Health Research Ethics (N-20220003).

### Test sessions

On day one, the cyclist reported to the laboratory for test session one. A full body dual energy x-ray absorptiometry (DEXA) scan (GE Lunar iDXA, GE Medical Systems, Madison, Wisconsin, USA) was carried out to determine body composition. In case that the cyclist had the DEXA-scan performed in the morning, instructions were to report to the laboratory fasting. In case that the cyclist had the scan during the daytime, instructions were to eat and drink the same before pre- and postscans. The GE Lunar iDXA was calibrated prior to each test according to the manufacturer's guidelines.

**Figure 1:**
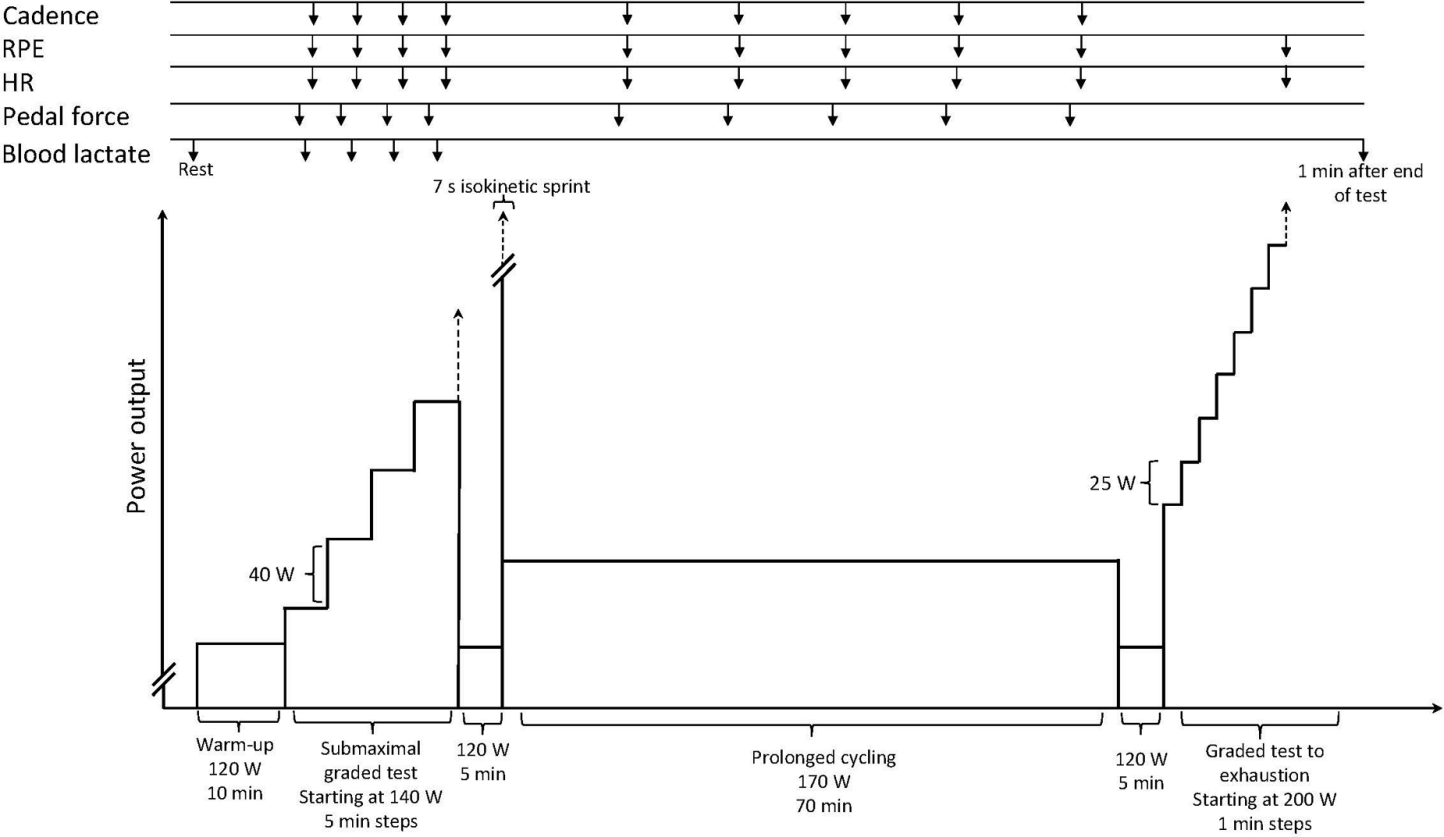
Illustration of test session two, which was applied at both pre- and posttest. The session contained several uninterrupted bouts of cycling and tests for measurement of performance indicators. Included is an overview of time points for sampling of diverse variables.

On a separate day, the cyclist reported to the laboratory for test session two (Figure 1). In advance, the cyclist was instructed to note the content of the two latest meals consumed prior to testing. Furthermore, the cyclist was instructed to consume the same meals prior to posttesting. In addition, the cyclist was instructed to refrain from intense exercise 48 h before testing. Test session two consisted of a cycling test conducted on an SRM cycle ergometer (Schoberer Rad Messtechnik, Jülich, Germany). Upon arrival, body mass (Seca gmbh & co., Hamburg, Germany) and height were measured and the temperature in the laboratory was noted. Next, the cycle ergometer was adjusted to the cyclist's preferences and the power output measuring unit was reset. The cyclist used his own pedals and shoes for the test. Next, a detailed presentation of the test protocol, including the method for blood sample collection (capillary blood drops taken from the cyclist's fingertip), was given. Subsequently, the participant's [Lā] was measured at rest, using a hand-held Accutrend Plus meter and BM-lactate test strips (Roche Diagnostics International AG, Rotkreuz, Switzerland). Performance checks, using BM-Control-Lactate solution, were made regularly, according to the manufacturer's recommendations.

Hereafter, a 10-min warm-up at a power output of 120 W was initiated. In direct prolongation of the warm-up, a submaximal graded test was initiated for determination of [Lā] at different power outputs (i.e., for creation of a lactate profile). The submaximal graded test started at 140 W and power output was increased by 40 W every 5 min until [Lā] reached a value of 4 mmol L^−1^ or above. Blood was sampled after 4 min of each bout and analysed for [Lā] during the remaining min of the bout. Of note is that the number of “steps” and applied power output values from the pre-test were repeated in the post-test, regardless of blood lactate concentration values in the post-test. The power output at a predetermined [Lā] of 2.5 mmol L^−1^ [W_2.5(Lā)_] was subsequently calculated by plotting [Lā] as a function of power output and applying linear regression between the two data points, which included the value of 2.5 mmol L^−1^. This method has previously been used to determine power output at predetermined values of [Lā] (4, 12). Pedal force was measured, throughout 30 s, at each bout. For details of the analysis of pedal force, please see below. During the 30 s pedal force recordings, the cyclist was instructed to remain seated in the saddle. In addition, cadence (in revolutions per minute, rpm), heart rate (HR, in beats per minute), and rating of perceived exertion (RPE) (13) were measured during the last min of each bout.

After completion of the submaximal graded test, the power output was reduced to 120 W, for 5 min. During these 5 min, the cyclist was given instructions about the approaching 7 s seated sprint at 80 rpm in which peak power output (W_peak_) was measured. The instruction was to perform a seated sprint with maximal effort for 7 s. Thirty s before the sprint they were instructed to pedal at a cadence as close to 77 rpm as possible and subsequently initiate the sprint from that starting point. For this test, the “isokinetic mode” (set to 80 rpm) of the cycle ergometer was applied. Verbal encouragement was given during the sprint. W_peak_ was determined as the highest power output value (analysed in windows of 0.5 s) obtained during the 7 s sprint. For all other cycling, the “constant power output mode” of the SRM cycle ergometer and “gear 8” was applied. In this mode, the preset power output is maintained by the ergometer regardless of applied cadence.

In direct continuation of the sprint, a bout of prolonged cycling for 70 min at 170 W was performed. Cadence, HR, and RPE were measured five times during the 70 min. The prolonged cycling was followed by 5 min of cycling at 120 W.

Finally, a graded test to exhaustion was performed for determination of maximal aerobic power output (W_max_). The test began with cycling for 1 min at 200 W. Thereafter, power output was increased by 25 W every min. The maximal incremental test ended when the cyclist reached voluntary exhaustion, or when the cyclist was unable to maintain a cadence of 60 rpm. HR was registered at the end of the test. RPE was noted immediately after the test was ended. [Lā] was measured one min after termination of pedalling. In case that the cadence dropped to less than 60 rpm, the cyclist was warned and given a last chance to increase the cadence. If the cyclist managed to do this within 5 s, the test continued. Verbal encouragement was given for this test. Maximal aerobic power output (W_max_, *y*) was calculated according to a previously applied procedure (14, 15) as: y=x+((a/60s)25W) where *x* is the second to last commenced power output and *a* is s of cycling at the last commenced power output.

The cyclist was allowed to consume water *ad libitum* and to stand up when necessary during the test. Furthermore, the cyclist was instructed to use freely chosen cadence throughout the test (except during the isokinetic seated sprint). The cyclist was given the opportunity to have a fan circulating the air in the room. The cyclist was blinded to cadence, except during tests for measurement of W_peak_ and W_max_. Furthermore, the cyclist was blinded to HR, throughout the test.

### Training period

The twelve-week period began at the start of the cycling season when the cyclists performed nearly all of their training outdoors. The cyclists in INT were instructed to perform maximal acceleration training on their own bicycle, at least three times per week, throughout the twelve-week period. The maximal acceleration training was a supplement to their usual training and consisted of a series of ten accelerations. Each acceleration consisted of a total of twenty pedal thrusts (i.e., ten with each leg) and was performed at maximal effort. Two min active rest separated the accelerations. The duration of a single acceleration was approximately 10 to 15 s and the total duration of a series of ten accelerations was approximately 22 min. The cyclist was instructed to perform the accelerations on a slightly ascending road, if possible, or on a horizontal road. Further, to maintain a seated position on the bike and apply one of the two largest gear ratios. The speed should be reduced to <5 km h^−1^ before initiating an acceleration. Altogether, this resulted in an initial low cadence and high resistance. The cyclist was furthermore instructed to, preferably, perform the maximal acceleration training at the beginning of the training session, after a warm-up. A video demonstration of the maximal acceleration training, including instructions, was provided for the cyclist. The cyclists in CON performed their usual cycling training throughout the twelve-week period. As for the “usual” part of the training, this was not influenced by the researchers.

### Training diary

A weekly training diary was collected from each cyclist. The diary contained information about training frequency and duration. In addition, it contained an overall RPE score for each training session. The score was based on a 10-point session RPE scale (16). According to the scale, 1 corresponds to “very, very easy”, 2 to “easy”, 3 to “moderate”, 4 to “somewhat hard”, 5 to “hard”, 7 to “very hard”, and 10 to “maximal”. Training load for each session, in an arbitrary unit, was subsequently estimated as follows:

Trainingload=durationoftrainingsession(min)×sessionRPE

(16). Subsequently, an average training load was calculated across the week and eventually an average across all weeks, for each cyclist. These values were used for further analysis. The method for training load determination by Foster et al. (2001) has been described as valid and reliable within a broad range of sports, including cycling (17). Cyclists in INT, furthermore, reported the number of accelerations performed each week.

### Data collection and analysis

Power output and cadence were measured by the SRM cycle ergometer at a sampling rate of 2 Hz. The exact points in time for noting the cadence during cycling can be seen in Figure 1.

Tangential and radial pedal forces from left and right pedal were recorded by a PowerForce System (Radlabor GmbH, Freiburg, Germany). During the submaximal graded test, 30-s pedal force recordings were made from 2:30 to 3:00 min in each bout. During the bout of prolonged cycling, pedal force recordings were made for 30 s at 14, 28, 42, 56, and 70 min (Figure 1). Pedal forces were recorded at 1,000 Hz using a 16-bit A/D converter and the data acquisition LabVIEW-based software IMAGO Record (part of the Powerforce system). For each 30 s pedal force recording, the Powerforce system calculated single mean pedal force profiles for one revolution, for each of the two pedals, and for each of the two pedal forces (i.e., four profiles). From these profiles, nine key characteristics were extracted. These characteristics include maximal tangential pedal force (Ft_max_), minimal tangential pedal force (Ft_min_), maximal radial pedal force (Fr_max_), minimal radial pedal force (Fr_min_), as well as the crank angles at which these values occurred. Furthermore, the length of the phase of negative tangential pedal force (Ph_neg_), measured in degrees, was extracted. Mean values from the left and the right pedal were calculated for each of these values, before further analysis. This procedure of analysis has previously been applied (18, 19). For examples of a tangential and radial pedal force profiles, the reader is referred to previous publications (19, 20).

HR was measured by a Garmin ForeRunner 245 (Garmin International Inc., Olathe, KS, USA) and noted at different time points throughout the test (Figure 1). For each HR determination during the submaximal graded test and during the bout of prolonged cycling, the value was calculated as the average of two readings, separated by 10 s. Regarding the graded test to exhaustion, HR was noted at the end of the test.

### Statistical analysis

All data are presented as mean ± SD, unless otherwise indicated. Test for normality (Shapiro-Wilks) was performed in IBM SPSS 27.0 (IBM Corporation, IBM SPSS Statistics, Armonk, NY, USA). Nonparametric data were tested using Wilcoxon signed-rank tests in SPSS. It is noted in the results section when non-parametric statistics was applied. Parametric data were tested using Student's *t* tests in Microsoft Excel 2019 (Microsoft Corporation, Bellevue, WA, USA) and repeated measures ANOVAs in SPSS. *p* ≤ 0.050 was considered to indicate a statistically significant difference.

## Results

### Performed training

INT and CON performed on average 4.3 ± 1.1 and 4.4 ± 2.1 training sessions per week during the twelve-week period, respectively (*p = *0.912). Regarding time spent on training, INT and CON performed on average 9.1 ± 3.2 and 9.3 ± 2.7 h of cycling per week (*p = *0.893). Regarding training load, INT and CON had a weekly training load of 3145 ± 1,162 and 3,066 ± 667 during the training period, respectively (*p = *0.841). INT performed on average 30.2 ± 4.2 (range: 25 to 38) accelerations per week during the performed supplementary maximal acceleration training.

### Temperature and time of day for testing

The temperature in the laboratory was 23.7 ± 1.2 °C and 25.7 ± 0.8 °C at the pre- and posttest, respectively (*p < *0.001). Of note is that there were no differences in temperature between the groups at each point in time (*p* > 0.050). For INT, the time of day for the DEXA-scan differed by 2 ± 2 h between the pre- and posttest. For CON, the time of day for the DEXA-scan differed by 0 ± 1 h between the pre- and posttest. For INT, the time of day for the cycling test differed by 0 ± 0 h between the pre- and posttest. For CON, the time of day for the cycling test differed by 1 ± 2 h between the pre- and posttest.

### Body mass, fat percentage, fat-free mass, and bone mass

Body mass for INT was 79.7 ± 12.2 kg and 78.7 ± 10.9 kg at the pre- and posttest, respectively. For CON, the body mass was 82.6 ± 11.0 and 80.3 ± 10.0 kg at the pre- and posttest, respectively. There was no difference between the groups at the pretest (*p = *0.537). For INT, the relative difference between the pre- and posttest was −0.9 ± 2.9%. For CON, the relative difference between the pre- and posttest was −2.7 ± 4.1%. The relative difference was not significantly different between groups (*p = *0.242).

Fat percentage for INT was 19.8 ± 6.5% and 19.0 ± 6.2% at the pre- and posttest, respectively. For CON, fat percentage was 23.4 ± 5.5% and 20.6 ± 4.8% at the pre- and posttest, respectively. There was no difference between the groups at the pretest (*p = *0.153). For INT, the difference between the pre- and posttest was −0.7 ± 2.5 percentage points. For CON, the difference between the pre- and posttest was −2.8 ± 2.6 percentage points. These values were significantly different (Wilcoxon signed-rank test, *p = *0.037).

Fat-free mass for INT was 59.9 ± 5.8 kg and 59.7 ± 6.0 kg at the pre- and posttest, respectively. For CON, fat-free mass was 59.3 ± 5.3 kg and 59.9 ± 5.0 kg at the pre- and posttest, respectively. There was no difference between the groups at the pretest (*p = *0.807). For INT, the relative difference between the pre- and posttest was −0.2 ± 1.6%. For CON, the relative difference between the pre- and posttest was 1.1 ± 2.4%. These values were not significantly different (*p = *0.117).

Bone mass for INT was 3.15 ± 0.49 kg and 3.15 ± 0.47 kg at the pre- and posttest, respectively. For CON, bone mass was 3.04 ± 0.36 kg and 2.99 ± 0.36 kg at the pre- and posttest, respectively. There was no difference between the groups at the pretest (*p = *0.542). For INT, the relative difference between pre- and posttest was 0.1 ± 1.2%. For CON, the relative difference between pre- and posttest was −1.7 ± 1.7%. These values were significantly different (Wilcoxon signed-rank test*, p = *0.028).

### W_2.5[lā]_ and supplementary data

W_2.5[Lā]_ was not different between the groups at the pretest (*p = *0.621). Further, there was no difference between the groups in the relative change in W_2.5[Lā]_ from pre- to posttest (*p = *0.351). The reader is referred to Table 1 for values of W_2.5[Lā]_.

**Table 1:** Performance indicators before (pretest) and after (posttest) a 12-week period of training. In addition, relative changes (*Δ*) from pretest to posttest are included. Values are presented as mean ± SD.

	Pretest (W)	Posttest (W)	*Δ* (%)
W_2.5[Lā]_			
INT	232.9 ± 46.1	240.8 ± 35.7	5.7 ± 17.5
* *CON	224.5 ± 35.2	221.1 ± 48.6	−1.1 ± 17.4
W_max_			
* *INT	370.9 ± 42.2	385.6 ± 32.3	4.5 ± 8.5
* *CON	361.4 ± 36.7	377.5 ± 36.4	4.8 ± 8.3
W_peak_			
INT	903.3 ± 158.4	933.7 ± 139.7	4.1 ± 8.0^a^
CON	923.7 ± 123.4	895.1 ± 116.1	−2.8 ± 7.7

INT, group performing supplementary maximal acceleration training. CON, control group performing usual training. W_2.5[Lā]_, power output at a fixed blood lactate concentration of 2.5 mmol L^−1^. W_max_, maximal aerobic power output obtained in a graded test to exhaustion. W_peak_, peak power output obtained in an isokinetic sprint.

^a^ Different from CON (*p* = 0.045).

HR during the submaximal graded test was similar for INT and CON, as well as for pre- and posttest, and consequently collapsed for an analysis of merely the effect of power output. HR at 140 W, 180 W, 220 W, 260 W, 300 W, 340 W, and 380 W was 107.8 ± 9.2 (n = 24), 119.9 ± 10.9 (*n* = 24), 133.0 ± 12.6 (*n* = 24), 146.3 ± 13.4 (*n* = 21), 156.2 ± 12.5 (*n* = 16), 165.3 ± 9.8 (*n* = 6), and 169.0 ± 0.0 (*n* = 1) beats per min, respectively. There was a significant effect of power output on HR for the three lowest power outputs (repeated measures ANOVA, *F = *410.763*; p < *0.001).

RPE during the submaximal graded test was similar for INT and CON, as well as for pre- and posttest, and consequently collapsed as HR. RPE at 140 W, 180 W, 220 W, 260 W, 300 W, 340 W, and 380 W was 8.6 ± 1.7 (*n* = 24), 10.1 ± 1.5 (*n* = 24), 11.9 ± 1.6 (*n* = 24), 13.8 ± 1.3 (*n* = 21), 15.4 ± 1.6 (*n* = 16), 15.8 ± 1.1 (*n* = 6), and 17.0 ± 0.0 (*n* = 1), respectively. There was a significant effect of power output on RPE for the three lowest power outputs (repeated measures ANOVA, *F* = 103.489; *p < *0.001).

Cadence and pedal force characteristics during the submaximal graded test were similar for INT and CON, as well as for pre- and posttest, and consequently collapsed as HR. Data for cadence and tangential pedal force variables are presented in Table 2. Briefly, there was a significant effect of power output on Ft_max_, Ft_min_, crank angle at Ft_max_, and Ph_neg_ for the three lowest power outputs (repeated measures ANOVA, *p < *0.050). Regarding radial pedal force variables, the following applied. Fr_max_ at 140 W, 180 W, 220 W, 260 W, 300 W, 340 W, and 380 W was 234 ± 36 N (*n* = 24), 262 ± 42 N (*n* = 24), 287 ± 44 N (*n* = 24), 312 ± 41 N (*n* = 21), 352 ± 52 N (*n* = 16), 354 ± 62 N (*n* = 6), and 455 ± 0.0 N (*n* = 1), respectively. There was a significant effect of power output on Fr_max_ for the three lowest power outputs (repeated measures ANOVA, *F = *209.447*; p < *0.001). Fr_min_ at 140 W, 180 W, 220 W, 260 W, 300 W, 340 W, and 380 W was −48 ± 22 N (*n* = 24), −54 ± 27 N (*n* = 24), −59 ± 26 N (*n *= 24), −66 ± 24 N (*n* = 21), −76 ± 34 N (*n* = 16), −83 ± 26 N (*n* = 6), and −55 ± 0.0 N (*n* = 1), respectively. There was a significant effect of power output on Fr_min_ for the three lowest power outputs (repeated measures ANOVA, *F = *18.263; *p < *0.001). Crank angle at Fr_max_ at 140 W, 180 W, 220 W, 260 W, 300 W, 340 W, and 380 W was 146 ± 15° (*n* = 24), 141 ± 14° (*n* = 24), 136 ± 13° (*n* = 24), 133 ± 14° (*n* = 21), 134 ± 15° (*n* = 16), 124 ± 17° (*n *= 6), and 151 ± 0.0° (*n* = 1), respectively. There was a significant effect of power output on the crank angle at Fr_max_ for the three lowest power outputs (repeated measures ANOVA, *F = *9.094; *p = *0.003).

**Table 2:** Cadence and selected tangential pedal force profile characteristics during the submaximal graded test for measurement of power output at a blood lactate concentration of 2.5 mmol L^−1^ [W_2.5(Lā)_]. Data were collapsed for the two groups of cyclists as well as for pretest and posttest. Values are presented as mean ± SD.

Power output (W)	*n*	Cadence (rpm)	Ft_max_^a^ (N)	Ft_min_^a^ (N)	Crank angle at Ft_max_^a^ (◦)	Crank angle at Ft_min_ (◦)	Ph_neg_^a^ (◦)
140	24	85.1 ± 9.9	253 ± 33	−72 ± 18	92 ± 7	268 ± 12	144 ± 12
180	24	84.7 ± 10.1	296 ± 38	−65 ± 20	89 ± 6	268 ± 14	138 ± 14
220	24	85.1 ± 9.9	332 ± 39	−57 ± 22	88 ± 5	265 ± 16	128 ± 20
260	21	84.3 ± 10.3	370 ± 44	−51 ± 23	87 ± 5	268 ± 20	117 ± 22
300	16	81.8 ± 10.7	416 ± 51	−48 ± 25	86 ± 6	277 ± 20	108 ± 28
340	6	80.0 ± 11.5	448 ± 86	−30 ± 9	83 ± 7	283 ± 21	93 ± 25
380	1	93.0	434	−28	79	265	89

Ft, tangential pedal force; Ph_neg_, phase with negative values of Ft.

^a^ Significant effect of power output for the three lowest power outputs (repeated measures ANOVA, *p* < 0.050). SD-values at 380 W are not meaningful and, therefore, not included.

### W_max_ and supplementary data

W_max_ was not different between the groups at the pretest (*p = *0.562). Further, there was no difference between the groups in the relative change in W_max_ from pre- to posttest (*p *= 0.943). See Table 1 for W_max_ values. As relative value, W_max_ was overall 4.63 ± 0.99 W kg^−1^.

HR_max_ for INT was 181.1 ± 11.9 and 178.8 ± 9.2 beats per min at the pre- and posttest, respectively. For CON, HR_max_ was 177.4 ± 11.1 and 174.9 ± 13.4 beats per min at the pre- and posttest, respectively. There was no difference between the groups at the pretest (*p = *0.444). For INT, the relative difference between the pre- and posttest was −1.1 ± 4.0%. For CON, the relative difference between pre- and posttest was −1.5 ± 2.7%. These values were not different (*p = *0.788).

RPE for INT, at the end of the test, was 18.9 ± 0.5 and 19.1 ± 0.7 at the pre- and posttest, respectively. For CON, RPE at the end of the test was 19.2 ± 0.7 and 19.2 ± 0.9 at the pre- and posttest, respectively. There was no difference between the groups at the pretest (Wilcoxon signed-rank test, *p = *0.317). For INT, the relative difference between the pre- and posttest was 0.9 ± 3.9%. For CON, the relative difference between the pre- and posttest was 0.0 ± 3.9%. These values were not different (Wilcoxon signed-rank test, *p = *0.719).

[Lā] for INT, one min after ending the test, was 8.3 ± 2.8 and 7.8 ± 3.5 mmol L^−1^ at the pre- and posttest, respectively. For CON, [Lā] one min after ending the test was 7.9 ± 2.3 and 9.2 ± 3.4 mmol L^−1^ at the pre- and posttest, respectively. There was no difference between the groups at the pretest (*p = *0.677). For INT, the relative difference between the pre- and posttest was −1.4 ± 37.3%. For CON, the relative difference between the pre- and posttest was 22.9 ± 48.3%. These values were not different (*p = *0.182).

### W_peak_

W_peak_ was not different between the groups at the pretest (*p = *0.728). The relative difference in W_peak_ from pre- to posttest was significantly larger for INT compared to CON (*p = *0.045). See Table 1 for W_peak_ values.

### Cadence, HR, and RPE during prolonged cycling

Two cyclists gave notice of the intensity being too strenuous during the prolonged cycling at the pretest. To make sure that these cyclists could complete the test, power output was reduced. One cyclist had the power output reduced to 160 W after 28 min. The other cyclist had the power output reduced to 160 W after 30 min and further reduced to 155 W after 43 min. Importantly however, identical procedures were applied at the posttest for these two cyclists.

In general, for cadence, HR, and RPE during the prolonged cycling, data were similar for INT and CON, as well as for the pre- and posttest. Consequently, data were collapsed to merely analyse for an effect of time. Cadence at 14 min, 28 min, 42 min, 56 min, and 70 min was 82.9 ± 8.9 rpm, 82.5 ± 8.8 rpm, 82.1 ± 9.7 rpm, 81.7 ± 9.5 rpm, and 81.2 ± 9.5 rpm, respectively. There was no effect of time on cadence (repeated measures ANOVA, *F = *1.173*; p = *0.322).

HR at 14 min, 28 min, 42 min, 56 min, and 70 min was 127.6 ± 9.6, 126.4 ± 10.3, 124.7 ± 11.0, 125.1 ± 11.0, and 126.3 ± 11.5 beats per min, respectively. There was a significant effect of time on HR (repeated measures ANOVA, *F = *4.739*; p = *0.014).

RPE at 14 min, 28 min, 42 min, 56 min, and 70 min was 10.2 ± 1.7, 10.7 ± 2.0, 11.0 ± 1.9, 11.1 ± 1.8, and 11.3 ± 1.8, respectively. There was a significant effect of time on RPE (repeated measures ANOVA, *F = *14.993*; p < *0.001).

## Discussion

The maximal acceleration training had a training-specific effect. Thus, W_peak_ in the 7-s maximal isokinetic sprint test was increased, about 4%, and to a statistically significantly larger extent for the cyclists who had performed the maximal acceleration training, as compared to the control group. Still, the effects of the applied maximal acceleration training were, overall, considered modest, as no effects were observed for W_2.5[Lā]_ and W_max_. It is possible that the series of brief maximal accelerations did not provide sufficient loading and stimulus for adaptations in additional performance indicators [W_2.5(Lā)_ and W_max_] to occur, as it has been reported for strength training (see Introduction). The maximal pedal force that can be produced during brief maximal-effort cycling at a low cadence of 30 rpm is about 1000 N (8). For comparison, about 1500 N can be produced by well-trained cyclists in a half-squat where the barbell is placed on the shoulders (5). A part of the reason for the difference in produced force is that the body is not pressed downwards during maximal accelerations on a bicycle.

An observation from the supplementary measurements during the submaximal graded test deserves a notion even that it is not of direct relevance to the hypothesis of the present study. The observation is that the freely chosen cadence was unaffected by power output during the submaximal graded test. One could intuitively expect that when the maximal tangential and radial pedal force is increased markedly, due to increased power output (as shown in Table 2 and described in Results), the cyclist would prefer to reduce the high pedal force by pedalling faster. However, this did not occur. The same has been reported for cycling on a Lode Excalibur Sport cycle ergometer (Lode B.V., Groningen, The Netherlands)(21) and for cycling on a Velodyne trainer (Schwinn Corp., Chicago IL)(22). These latter cycle ergometers are electromagnetically braked, like the applied SRM ergometer. Apparently, these cycle ergometers do not cause the cyclists to behave completely like in more natural conditions. Thus, during treadmill cycling (8, 23) and road cycling (24, 25), the freely chosen cadence increases with an increase in power output. It is possible that the dissimilar findings are related to the source of mechanical resistance that is applied (air, rolling, friction, and gravitational resistance vs. electromagnetically generated resistance). In general, the magnitude of crank inertial load (which is low for many cycle ergometers as compared with fast road or treadmill cycling), as well as the perceived exertion and motor control related to overcoming the crank inertial load and resistance (23, 26), may serve to explain this finding. A couple of other observations also deserve a notion. Thus, the changes of fat percentage as well as bone mass, across the study period, were statistically significantly different between the two groups. We have no other explanation for these results than that they might reflect random outcomes.

RPE increased across time during the prolonged cycling. This was the intention, since test session two was designed to, to an extent, simulate work demands in road cycling. Still, RPE merely increased by on average about 1 point on the Borg Scale. If a higher power output had been applied during the prolonged cycling, RPE would presumably have increased more. And perhaps this would have caused a different outcome. On the other hand, a couple of the participants had the power output reduced during the prolonged cycling, to be able to complete. This indicates that power output should not have been higher than the applied 170 W. An alternative would have been to individualize the intensity, based on work capacity. However, when relatively homogeneous participants are studied, as in the present study, it is meaningful to apply an absolute rather than a relative intensity during testing. Furthermore, an absolute intensity better resembles real world cycling for a group of cyclists that stays together at the same speed.

Strengths and limitations of the present study should be considered. For example, a largely ecological design was applied. This makes the transfer from research to real world cycling relatively easy. Several choices were made about various study design details in the planning phase of the study. Retrospectively these choices, combined with the present results, generate several questions that may be addressed in future studies. Had the results been similar if another category of cyclists was tested? Had a longer training period changed the results? Had another number of accelerations in each series and perhaps more series per week altered the results? Had measurement of other performance indicators (e.g., a sprint at the end of the prolonged cycling rather than the performed graded test to exhaustion) altered the conclusions? Answers to these questions are open for speculation.

The present study calls for some practical considerations. For example, the performed maximal acceleration training is easy to integrate as a part of an outdoor cycling training session. In addition, the present study showed a modest advantage of performing the maximal acceleration training, as regards to performance indicators. Even such a modest advantage may still be meaningful to achieve for competitive cyclists since marginal performance improvements can be decisive in a tight finish.

## Conclusion

The present study, which included trained cyclists, showed the following. Twelve weeks of training supplementation with maximal accelerations resulted in improvement in a single out of a collection of performance indicators, as compared to the change measured in a control group. Thus, peak power output in a 7-s maximal isokinetic sprint test was increased (by on average 4.1%) to a statistically significantly larger extent in the cyclists who supplemented their usual training with maximal accelerations as compared to the change observed for the cyclists in the control group. The changes in maximal aerobic power output as well as the power output at a blood lactate concentration of 2.5 mmol L^−1^, both determined in graded tests, were not different between the two groups.

## Data Availability

The raw data supporting the conclusions of this article will be made available by the authors, without undue reservation.
